# Protrusio Acetabuli in Osteogenesis Imperfecta: A Two-Center Study on Incidence and Associated Factors

**DOI:** 10.1007/s00223-026-01554-2

**Published:** 2026-06-02

**Authors:** Maria Gjættermann, Andrea Laufer, Jannie Dahl Hald, Jan Duedal Rölfing, Bjoern Vogt, Bjarne Møller-Madsen

**Affiliations:** 1 Department of Children’s Orthopaedics, Aarhus University Hospital, Aarhus, Denmark; 2 Danish Paediatric Orthopaedic Research, Aarhus University Hospital, Aarhus, Denmark; 3 Pediatric Orthopedics, Deformity Reconstruction and Foot Surgery, Muenster University Hospital, Muenster, Germany; 4 Department of Endocrinology and Internal Medicine, Aarhus University Hospital (AUH), Aarhus, Denmark; 5 Center for Rare Disorders, Department of Paediatrics, Aarhus University Hospital (AUH), Aarhus, Denmark; 6 Department of Clinical Medicine – Orthopaedic Surgery, Aarhus University, Aarhus, Denmark

**Keywords:** Protrusio acetabuli, Osteogenesis imperfecta, Hip deformity, Acetabular deformity, Sillence classification

## Abstract

Osteogenesis imperfecta (OI) is a rare genetic connective tissue disorder characterized by increased fracture risk and bone deformity. Protrusio acetabuli (PA) is a well-recognized radiographic finding in patients with OI, yet data on its incidence, severity, and associated factors across different OI phenotypes remain limited. This retrospective study evaluated pelvic radiographs and clinical data of patients with OI treated in two centers. PA was assessed using established radiographic criteria and graded according to the Sotelo-Garza/Charnley classification. Demographic and clinical variables were analyzed to identify factors associated with PA. Of 69 patients included, PA was found in 32 (46.4%), of whom nine presented with severe, 15 with moderate, and eight with mild PA. Patients with PA were significantly older than those without PA. Univariable logistic regression identified female sex, body mass index > 25 kg/m^2^, and scoliosis as factors significantly associated with PA. PA is a frequent radiographic finding in OI, affecting patients across all phenotypic severities, including mild disease, and may represent an underrecognized manifestation across the disease spectrum. While several factors were associated with its presence, these findings should be interpreted descriptively. Given the potential for selection bias, the reported prevalence may not be generalizable to the broader OI population. Further prospective studies are required to clarify the clinical relevance and natural history of PA in OI.

Level of Evidence IV.

## Introduction

Osteogenesis imperfecta (OI) is a rare, generalized connective tissue disorder with a wide phenotypic variety. Approximately 90% of the cases are caused by mutations in the genes *COL1A1* and *COL1A2,* which lead to alterations in encoding type I collagen, resulting in an abnormal or reduced quantity of produced collagen [6]. OI primarily affects the skeleton and is most commonly classified according to severity as originally proposed by Sillence [11, 17]. Common clinical manifestations include low bone mass, reduced body height, bone deformities and bone fragility resulting in an increased risk of pathological fractures [3, 4, 10, 15]. Extraskeletal manifestations such as blue sclerae, ligamentous laxity and cardiopulmonary and/or gastrointestinal complications may also be present [5]. The increased deformability of the pelvic bones in conjunction with reduced cortical thickness predisposes to medial acetabular protrusion [8, 13]. As soon as the acetabulum and femoral head have migrated medially to the ilioischial line, the rate of progression will increase into the pelvic cavity [7]. Few studies have yet investigated incidence and clinical consequences of PA in OI patients [1, 9, 15, 18].

This study aims to analyze the incidence and severity of PA in a cohort of patients with OI at two centers, and to evaluate the presence of PA depending on OI type. In addition, clinical manifestations potentially related to PA and factors associated with PA were explored descriptively to refine the understanding of PA in OI patients and to facilitate diagnosis and treatment.

## Patients and Methods

**Fig. 1:**
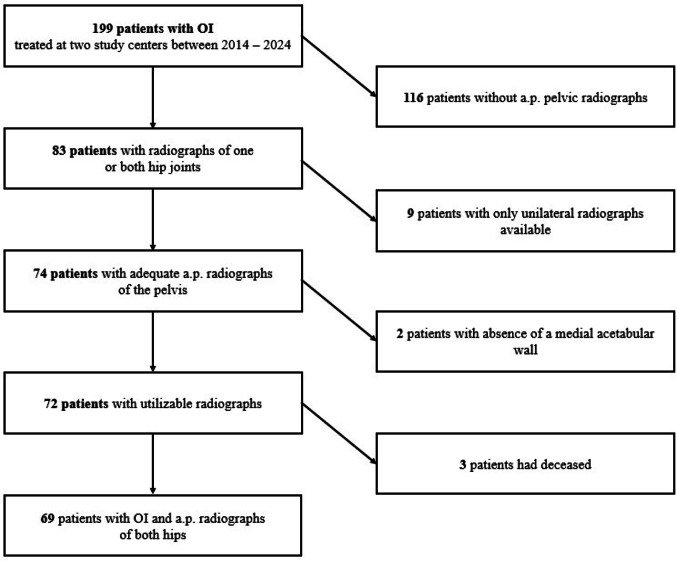
This STROBE diagram details the inclusion and exclusion criteria for the study cohort. *OI* Osteogenesis imperfecta, *a. p.* anteroposterior

The study is reported according to Strengthening the Reporting of Observational Studies in Epidemiology (STROBE) guidelines [19]. Medical records and pelvic radiographs of patients with a clinical and/or molecular genetic diagnosis of OI treated at two departments were retrospectively evaluated. At site one, the data collection was part of a quality study approved in 2023. At site two, data of patients treated between 2014 and 2023 were collected. At both sites, a total of 199 patients with OI were identified, of whom radiographs of 69 patients were ultimately available for analysis (Fig. 1). A comparison between included and non-included patients revealed differences in age distribution and OI severity. Included patients were younger, with a higher proportion of individuals under 18 years (52% vs. 21%), and showed a greater representation of moderate to severe OI phenotypes (types III and IV). In contrast, non-included patients were more frequently adults and more often classified as type I or had unknown subtype classification. These findings indicate a potential selection bias toward younger and more clinically affected patients with available radiographs.

All data, including the OI type based on the Sillence classification, sex, age, presence of spinal deformities or leg length discrepancy (LLD), information regarding reliance on mobility aids, and anthropometric measurements such as body mass index (BMI) were collected. Scoliosis was analyzed as a categorical variable (present/absent), as standardized Cobb angle measurements were not consistently available across the cohort. Bisphosphonate treatment was documented; however, due to the high prevalence of treatment across the cohort, this variable was not further analyzed. According to the Sillence classification, OI was categorized as mild (type I), moderate (type IV), and severe (type III and XI). If the OI type was not recorded, the subtype classification was performed based on clinical documentation, including phenotypic features, height, and skeletal radiographs. In 16 patients, subtype assignment was conducted retrospectively by two investigators through independent review of clinical and imaging data, followed by consensus. Genetic information was available for a subset of patients (28/69 with detailed genetic data and 5/69 with confirmed genetic diagnosis without specification); however, due to incomplete genetic data across the cohort, classification was primarily based on phenotypic Sillence criteria to ensure consistency. Mobility aids consists of a wheelchair, walker, or mobility scooter. These independent variables were collected during the same time period as the radiographs were performed to ensure a more accurate and valid representation of the condition. The variables were used to identify potential factors for developing PA. Medical records of all patients with radiographically verified PA were thoroughly examined to identify potential clinical complications associated with PA. In patients with multiple radiographs, the most recent image was selected to reflect the current skeletal status. If image quality was insufficient for reliable assessment of anatomical landmarks, the most recent radiograph with adequate quality was used instead.

**Fig. 2:**
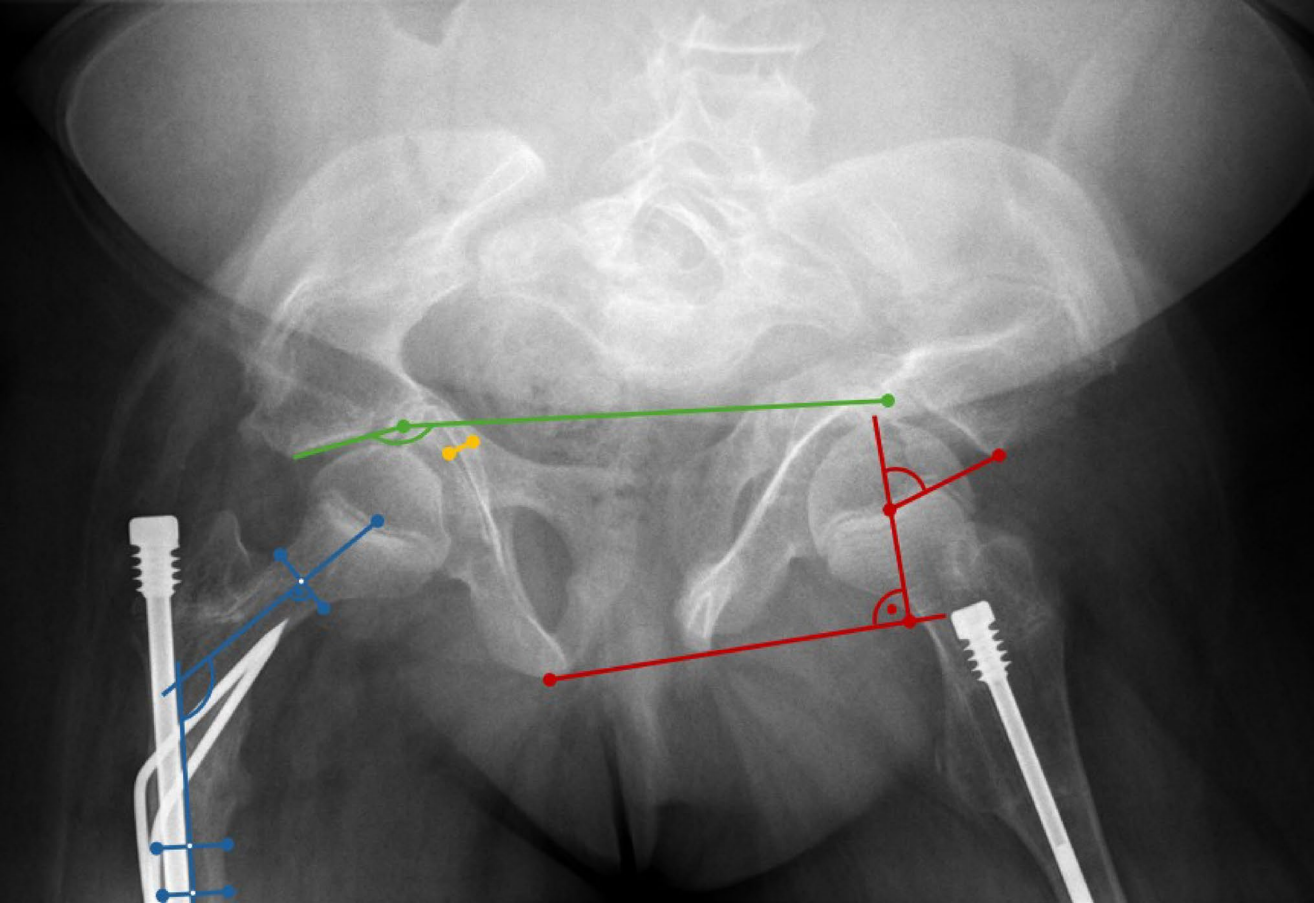
Anteroposterior (AP) radiograph of the pelvis illustrating the measurement of key radiographic parameters. The acetabular index is shown in green, demonstrating a negative value in this case. The center–edge angle (Wiberg angle) is indicated in red. The neck–shaft angle is depicted in blue. The distance between the medial acetabular wall and the ilioischial line, used for grading acetabular protrusion according to the Sotelo-Garza/Charnley classification, is marked in yellow

The radiographic criteria for identifying PA were previously established by Van de Velde et al. [16]. PA was diagnosed in one or both hips if at least two of the following parameters were present: (1) crossing of the acetabulum medial to the iliopectineal line, (2) crossing of the teardrop figure or intersection of the acetabulum medial to Koehler’s ilioischial line, (3) increased center-edge (CE) angle of Wiberg (Fig. 2) [16, 18]. PA was diagnosed in children when the medial wall of the acetabulum crossed the ilioischial line by > 3 mm in girls and by > 1 mm in boys, respectively. In adults, > 6 mm in women and > 3 mm in men, respectively, were determined as the threshold. The teardrop sign was regarded as diagnostic when crossing or reversing the ilioischial line [2, 16]. The CE angle threshold was determined as 35 degrees in children and 40 degrees in adults [16, 18].

The presence of PA in each OI patient was graded as either mild (< 5 mm), moderate (6–15 mm), or severe protrusion (> 16 mm) according to the Sotelo-Garza/Charnley classification [12]. All radiographic measurements were performed by a single investigator. To assess intra-rater reliability, each measurement was repeated on three occasions at intervals of at least one week, and intraclass correlation coefficients (ICC) were calculated using a two-way random-effects model with absolute agreement.

### Statistical Analysis

Statistical analyses were performed using R version 4.5.2 (R Foundation for Statistical Computing, Vienna, Austria). Categorical variables are presented as absolute numbers and percentages. Continuous variables are reported as mean ± standard deviation (SD) if normally distributed, or as median with interquartile range (IQR; 25th–75th percentile) if not normally distributed.

Comparisons between groups were performed using the independent-samples *t* test for normally distributed continuous variables and the Mann–Whitney U test for non-normally distributed continuous variables. Categorical variables were compared using the χ^2^ test or Fisher’s exact test, as appropriate.

Univariable logistic regression analysis was performed to estimate odds ratios (OR) with 95% confidence intervals (CI). A two-sided *p* value < 0.05 was considered statistically significant.

## Results

The median age of patients in this cohort was 18 years (IQR 12–33), comprising 32 male and 37 female patients. The distribution of patients according to the Sillence classification is presented in Table 1.

**Table 1:** Patient demographics by osteogenesis imperfecta type

OI type*	*n*	Age (y) (median with IQR)	M/F	Incidence of PA *n* (%)
I	24	17.0 (11.8–45.3)	14/10	11 (45.8)
III	12	16.5 (13.8–25.5)	6/6	8 (66.7)
IV	29	23.0 (14.0–33.0)	10/19	12 (41.4)
V	1	10 (10)	0/1	–
VI	1	15 (15)	1/0	–
XI	1	11 (11)	0/1	1
XIII	1	24 (24)	1/0	–
Overall	69	18.0 (12.0–33.0)	32/37	32 (46.4)

*Based on phenotypic Sillence classification with inclusion of additional genetically defined subtypes (types V, VI, XI, XIII). *OI* Osteogenesis imperfecta. *n* Number, *y* years, *M/F* male/female, *PA* Protrusio acetabuli

PA was present in 32 of 69 (46.4%) OI patients (Table 2), of whom 11 (34.4%) presented with bilateral PA. Radiographic measurements demonstrated excellent intra-rater reliability for both CE angle measurements (ICC = 0.98, 95% CI 0.98–0.99) and medial migration measurements (ICC = 1.00, 95% CI 0.99–1.00), indicating excellent measurement reproducibility.

**Table 2:** Data of OI patients presenting with PA

	Sillence type				Overall
	I	III	IV	XI	
OI patients with PA (*n*)	11	8	12	1	32
Age (*y*) Median (IQR)	18.0 (15.0–41.0)	20.5 (15.5–25.5)	27.0 (15.8–45.5)	11	22.5 (14.8–33.3)
Age ≥ 18 years (*n*)	7	4	8	–	19
Gender (M/F) (*n*)	4/7	3/5	2/10	0/1	9/23
BMI > 25^†^ *n* (%)	3 (27.3)	5 (62.5)	9 (75)	1 (100)	18 (56.3)
LLD > 2 cm *n* (%)	3 (27.3)	2 (25)	5 (41.7)	– (–)	10 (31.3)
Scoliosis *n* (%)	5 (45.5)	7 (87.5)	7 (58.3)	1 (100)	20 (62.5)
Mobility aids *n* (%)	2 (18.2)	7 (87.5)	7 (58.3)	– (–)	16 (50)
Constipation *n* (%)	1 (9.1)	5 (62.5)*	1 (8.3)	1 (100)	8 (25)
Bilateral PA *n* (%)	3 (27.3)	3 (37.5)	4 (33.3)	1 (100)	11 (34.4)

*OI* Osteogenesis imperfecta, *PA* Protrusio acetabuli, *n* number, *y* years, *M/F* male/female, *BMI* body mass index, *LLD* leg length discrepancy

*1 patient with colostomy

^†^Missing BMI values for two individuals were estimated as >25 kg/m^2^ based on imaging and clinical notes describing obesity

Among patients with PA, 15 reported pain in the hip, knee, or lower back and/or functional limitations potentially related to PA, whereas in other cases PA was identified as an incidental radiographic finding without documented clinical consequences. Due to heterogeneous clinical documentation, a standardized assessment of symptom severity was not possible. Sixteen patients required mobility aids (wheelchair, walker, or mobility scooter), while the remainder were ambulatory.

Four of 69 patients had undergone ipsilateral or bilateral total hip replacement. Overall, 42 of 69 patients had a history of surgery involving the hip or proximal femur, including intramedullary nailing or Fassier–Duval rodding for femoral shaft fractures (n = 29), intramedullary hip screw fixation for proximal femur fractures (n = 9), and total hip arthroplasty or hemiarthroplasty (n = 4). These procedures primarily reflected fracture-related interventions rather than treatment of PA.

**Fig. 3:**
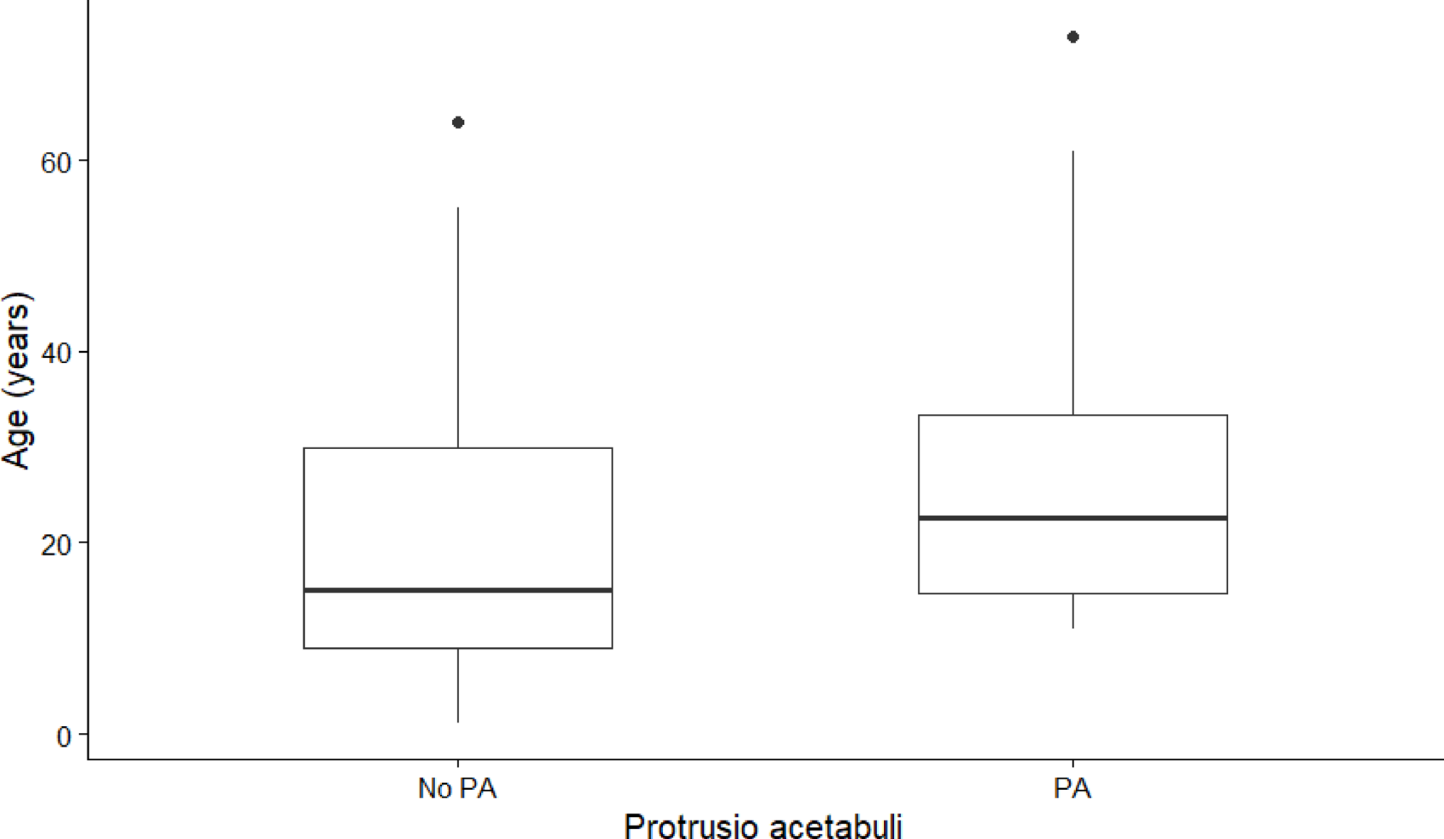
Boxplots comparing age distribution between patients with and without Protrusio acetabuli. The box represents the interquartile range with the median indicated by the horizontal line; whiskers extend to 1.5 × IQR, and points indicate outliers. *PA:* Protrusio acetabuli

PA was observed in 62.2% of female and 28.1% of male OI patients. Patients with PA were significantly older than those without PA (median 22.5 years, IQR 14.8–33.2 vs. 15.0 years, IQR 9.0–30.0; *p* = 0.037) (Fig. 3). Nineteen of 32 (59.4%) patients with PA were older than 18 years, and 23 (71.9%) were female.

The incidence of PA was highest among patients diagnosed with severe OI type III (66.7%). Nine patients were classified with mild protrusion according to the Sotelo-Garza/Charnley classification, 15 with moderate protrusion, and eight with severe protrusion. In one female patient with Sillence type III, the acetabulum was observed to progress medially to the iliopectineal line.

**Fig. 4:**
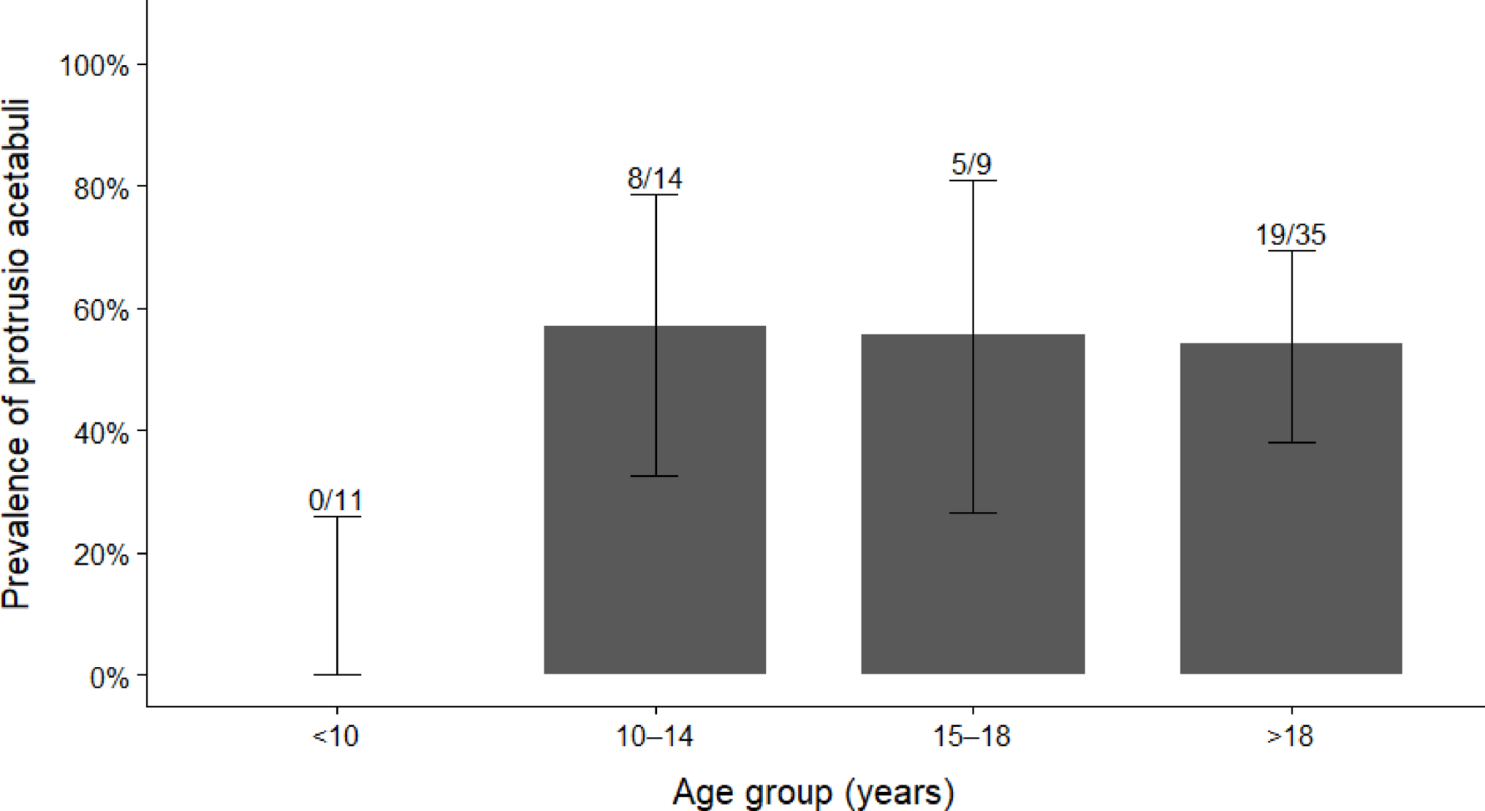
Age-stratified prevalence of Protrusio acetabuli (PA) in Osteogenesis imperfecta. Bar plot showing the proportion of patients with radiographic PA across predefined age groups (< 10, 10–14, 15–18, > 18 years). Error bars represent 95% confidence intervals for the prevalence estimates. Numbers above bars indicate the fraction of PA-positive patients over the total number in each age group (n/N)

Results of the univariable logistic regression analysis are presented in Table 3 and should be interpreted as exploratory associations rather than implying causality. In this analysis, female sex, BMI > 25 kg/m^2^, and scoliosis were statistically associated with PA, whereas age ≥ 18 years, LLD > 2 cm, and use of mobility aids were not (Table 3, Fig. 4).

**Table 3:** Univariable logistic regression analysis of exploratory associations with protrusio acetabuli

Variables	OR (95% CI)	*p* value
Age ≥ 18 years	1.92 (0.73–5.01)	0.183
Female gender	4.20 (1.52–11.61)	0.006
BMI > 25 kg/m^2^	3.04 (1.13–8.20)	0.028
LLD > 2 cm	1.23 (0.43–3.48)	0.7
Scoliosis	3.08 (1.15–8.23)	0.025
Mobility aids	1.85 (0.70–4.86)	0.21

*OR* odds ratio, *CI* confidence interval, *BMI* body mass index, *LLD* leg length discrepancy

## Discussion

PA is a radiographic finding frequently encountered in OI patients, which is associated with a higher susceptibility of fractures of the acetabulum and proximal femur due to increased mechanical stress [15]. In this retrospective two-center cohort, PA was observed in nearly half of OI patients who underwent radiographic evaluation. While PA was most frequent in individuals with moderate to severe phenotypes, a substantial proportion of patients with mild OI were also affected. This finding suggests that PA is not limited to advanced disease stages but may occur across the full clinical spectrum of OI.

The observed prevalence of 46% is consistent with previously reported rates ranging between 45 and 55% in selected OI cohorts [1, 15]. Lower prevalences reported in exclusively pediatric populations may reflect differences in age distribution and imaging indications [9, 18].

In the present study, patients with PA were significantly older than those without PA; however, age ≥ 18 years was not associated with PA in the regression analysis. Given the cross-sectional design, these findings should not be interpreted as evidence of age-dependent progression, as suggested by previous studies [1], but rather as differences in prevalence across age groups. This apparent discrepancy may reflect limited statistical power or collinearity with disease severity rather than the absence of a true age-related effect. Longitudinal studies are required to clarify the natural history and potential progression of PA in OI.

Female sex, BMI > 25 kg/m^2^, and the presence of scoliosis were associated with PA in univariable analyses. These findings are in line with previous reports, although their interpretation requires caution [1, 9]. Due to the retrospective and cross-sectional nature of the study, no causal relationships can be inferred. In addition, BMI may not fully reflect mechanical loading in OI, as reduced body height and skeletal deformities may limit its validity. Similarly, scoliosis was analyzed as a dichotomous variable and likely reflects overall disease severity rather than constituting an independent factor associated with acetabular medialization. The lack of association between PA and the use of mobility aids further supports the notion that these variables primarily capture disease burden rather than distinct mechanistic contributors.

The clinical relevance of PA in OI remains incompletely defined. In the present cohort, PA was identified both in symptomatic patients and as an incidental radiographic finding. While previous studies have suggested that PA may contribute to pain, reduced mobility, and, in severe cases, abdominal complications, our data do not allow for a systematic evaluation of these outcomes [9, 14]. Accordingly, the clinical implications of PA should be interpreted cautiously.

Several limitations should be acknowledged. The retrospective design and limited sample size preclude causal inference and multivariable adjustment. Radiographic assessment of PA is sensitive to pelvic positioning and skeletal deformities commonly present in OI, which may have introduced measurement bias. In addition, relevant radiographic parameters reflecting skeletal deformity were not systematically assessed, and advanced imaging such as 3D-CT was not available, potentially limiting differentiation between true and pseudo-protrusio deformities. Consequently, the reported incidence of PA may be overestimated, particularly in patients with severe pelvic deformity. Furthermore, only patients with available radiographs obtained based on clinical indication were included, introducing a risk of selection bias and potential overestimation of PA prevalence. This is supported by differences between included and non-included patients, suggesting that younger and more clinically affected individuals were more likely to undergo radiographic evaluation. These limitations should be considered when interpreting the reported findings of this study.

Despite these limitations, the present study adds to the limited body of literature on PA in OI by including both pediatric and adult patients across different phenotypic severities. The findings highlight that PA is frequently observed in radiographically evaluated OI patients and may occur even in clinically mild phenotypes. However, given the potential for selection bias and the descriptive nature of the analysis, further prospective studies are required to clarify the natural history and clinical relevance of PA in OI.

## Conclusion

In this retrospective two-center cohort study, PA was frequently observed among OI patients undergoing radiographic evaluation, affecting nearly half of the cohort and occurring across all phenotypic severities, including mild disease. These findings suggest that PA may represent an underrecognized skeletal manifestation across the disease spectrum. Female sex, elevated BMI, and spinal deformity were associated with PA, although these associations should be interpreted descriptively given the cross-sectional design.

Considering the clinical impact that PA may have, increased clinical awareness may be warranted, particularly in symptomatic patients. However, given the potential for selection bias, the reported prevalence may not be generalizable to the broader OI population. Prospective longitudinal studies are required to better define the natural history and clinical relevance of PA in OI.

## Data Availability

The datasets used and/or analyzed during the current study are available from the corresponding author on reasonable request.

## References

[1] Ahn J, Carter E, Raggio CL et al (2019) Acetabular protrusio in patients with osteogenesis imperfecta: risk factors and progression. J Pediatr Orthop 39:e750–e754 31599861 10.1097/BPO.0000000000001051

[2] Dunlop CC, Jones CW, Maffulli N (2005) Protrusio acetabuli. Bull Hosp Jt Dis 62:105–114 16022223

[3] Forlino A, Marini JC (2016) Osteogenesis imperfecta. Lancet 387:1657–1671 26542481 10.1016/S0140-6736(15)00728-X PMC7384887

[4] Jain M, Tam A, Shapiro JR et al (2019) Growth characteristics in individuals with osteogenesis imperfecta in North America: results from a multicenter study. Genet Med 21:275–283 29970925 10.1038/s41436-018-0045-1 PMC6320321

[5] Marini JC, Forlino A, Bachinger HP et al (2017) Osteogenesis imperfecta. Nat Rev Dis Primers 3:17052 28820180 10.1038/nrdp.2017.52

[6] Marlowe A, Pepin MG, Byers PH (2002) Testing for osteogenesis imperfecta in cases of suspected non-accidental injury. J Med Genet 39:382–386 12070242 10.1136/jmg.39.6.382 PMC1735162

[7] Mcbride MT, Muldoon MP, Santore RF et al (2001) Protrusio acetabuli: diagnosis and treatment. J Am Acad Orthop Surg 9:79–88 11281632 10.5435/00124635-200103000-00002

[8] Nijhuis WH, Eastwood DM, Allgrove J et al (2019) Current concepts in osteogenesis imperfecta: bone structure, biomechanics and medical management. J Child Orthop 13:1–11 30838070 10.1302/1863-2548.13.180190 PMC6376438

[9] Ramos-Mejia R, Monterroza-Quintana F, Primomo C et al (2023) Acetabular protrusion in a cohort of patients with osteogenesis imperfecta evaluated in a pediatric hospital. J Pediatr Genet 12:48–52 36684543 10.1055/s-0041-1732476 PMC9848761

[10] Rauch F, Glorieux FH (2004) Osteogenesis imperfecta. Lancet 363:1377–1385 15110498 10.1016/S0140-6736(04)16051-0

[11] Sillence DO, Senn A, Danks DM (1979) Genetic heterogeneity in osteogenesis imperfecta. J Med Genet 16:101–116 458828 10.1136/jmg.16.2.101 PMC1012733

[12] Sotelo-Garza A, Charnley J (1978) The results of Charnley arthroplasty of hip performed for protrusio acetabuli. Clin Orthop Relat Res 132:12–18 679527

[13] Stasek S, Zaucke F, Hoyer-Kuhn H et al (2025) Osteogenesis imperfecta: shifting paradigms in pathophysiology and care in children. J Pediatr Endocrinol Metab 38:1–15 39670712 10.1515/jpem-2024-0512

[14] Stockwell E, Wallace M (2022) Obstructive constipation in two patients with severe Osteogenesis Imperfecta and acetabular protrusio. JAAOS Glob Res Rev. 10.5435/jaaosglobal-d-21-00194 10.5435/JAAOSGlobal-D-21-00194 PMC8735770 34982054

[15] Trehan SK, Morakis E, Raggio CL et al (2015) Acetabular protrusio and proximal femur fractures in patients with osteogenesis imperfecta. J Pediatr Orthop 35:645–649 25379829 10.1097/BPO.0000000000000343

[16] Van De Velde S, Fillman R, Yandow S (2006) Protrusio acetabuli in Marfan syndrome. History, diagnosis, and treatment. J Bone Joint Surg Am 88:639–646 16510833 10.2106/JBJS.E.00567

[17] Van Dijk FS, Sillence DO (2014) Osteogenesis imperfecta: clinical diagnosis, nomenclature and severity assessment. Am J Med Genet A 164A:1470–1481 24715559 10.1002/ajmg.a.36545 PMC4314691

[18] Violas P, Fassier F, Hamdy R et al (2002) Acetabular protrusion in osteogenesis imperfecta. J Pediatr Orthop 22:622–625 12198464

[19] Von Elm E, Altman DG, Egger M et al (2007) The Strengthening the Reporting of Observational Studies in Epidemiology (STROBE) statement: guidelines for reporting observational studies. Epidemiology 18:800–804 18049194 10.1097/EDE.0b013e3181577654

